# Biodegradation of an injectable treated dentin matrix hydrogel as a novel pulp capping agent for dentin regeneration

**DOI:** 10.1186/s12903-023-02831-4

**Published:** 2023-02-25

**Authors:** Ahmed A. Holiel, Hossam M. Mustafa, Eman M. Sedek

**Affiliations:** 1 Conservative Dentistry Department, Faculty of Dentistry, Alexandria University, Alexandria, Egypt; 2 Oral Biology Department, Faculty of Dentistry, Alexandria University, Alexandria, Egypt; 3 Dental Biomaterials Department, Faculty of Dentistry, Alexandria University, Alexandria, Egypt

**Keywords:** Biodegradation, Dentin matrix, Injectable scaffold, Dentin regeneration, Pulp capping

## Abstract

**Background:**

A novel injectable mixture termed treated dentin matrix hydrogel (TDMH) has been introduced for restoring dentin defect in DPC. However, no study evaluated its physiological biodegradation. Therefore, the present study aimed to assess scaffold homogeneity, mechanical properties and biodegradability in vitro and in vivo and the regenerated dentin induced by TDMH as a novel pulp capping agent in human permanent teeth.

**Methods:**

Three TDMH discs were weighted, and dry/wet ratios were calculated in four slices from each disc to evaluate homogeneity. Hydrogel discs were also analyzed in triplicate to measure the compressive strength using a universal testing machine. The in vitro degradation behavior of hydrogel in PBS at 37 °C for 2 months was also investigated by monitoring the percent weight change. Moreover, 20 intact fully erupted premolars were included for assessment of TDMH in vivo biodegradation when used as a novel injectable pulp capping agent. The capped teeth were divided into four equal groups according to extraction interval after 2-, 8-, 12- and 16-weeks, stained with hematoxylin–eosin for histological and histomorphometric evaluation. Statistical analysis was performed using F test (ANOVA) and post hoc test (*p* = 0.05).

**Results:**

No statistical differences among hydrogel slices were detected with (*p* = 0.192) according to homogeneity. TDMH compression modulus was (30.45 ± 1.11 kPa). Hydrogel retained its shape well up to 4 weeks and after 8 weeks completely degraded. Histological analysis after 16 weeks showed a significant reduction in TDMH area and a simultaneous significant increase in the new dentin area. The mean values of TDMH were 58.8% ± 5.9 and 9.8% ± 3.3 at 2 and 16 weeks, while the new dentin occupied 9.5% ± 2.8 at 2 weeks and 82.9% ± 3.8 at 16 weeks.

**Conclusions:**

TDMH was homogenous and exhibited significant stability and almost completely recovered after excessive compression. TDMH generally maintained their bulk geometry throughout 7 weeks. The in vivo response to TDMH was characterized by extensive degradation of the hydrogel and dentin matrix particles and abundant formation of new dentin. The degradation rate of TDMH matched the rate of new dentin formation.

*Trial registration*: PACTR201901866476410: 30/1/2019.

## Background

Nowadays, regenerative dentistry is a promising area for dental tissue regeneration. Teeth affected by dental caries, traumatic dental injuries or even iatrogenesis could be benefited by this field advancing. Direct pulp capping (DPC), as an important treatment procedure for pulp exposure, requires adequate protection of the exposed pulp tissue without sacrificing its vitality and functions [1]. Regenerative dentistry based on tissue engineering allows to maintain not only structure of dentin-pulp complex but also its function, by stimulating dentinogenesis and restoring healthy tissues. The strategy focuses on creating scaffold-cell construct for inducing dental tissue regeneration [2, 3].

Many materials have been used to create scaffolds for dentin regeneration in DPC procedure. However, few have succeeded in obtaining complete dentin tissue [1]. It may be the case that while these materials support cell growth and mineralization, they are not capable of inducing differentiation towards an odontogenic specialization [4]. In order to achieve the perfect imitation for dentin regeneration, scaffold derived from dentin tissue is the best choice. A novel injectable mixture termed Treated Dentin Matrix Hydrogel (TDMH) combining alginate hydrogel as the matrix phase and TDM powder has been introduced as a promising material for restoring dentin defect in DPC [5]. It is not only a potential material satisfying the physical and chemical characteristics of a standard scaffold, but also the demand inductive factors for dentin-pulp complex regeneration [6–9]. As a result, TDMH based scaffold facilitated dentinogenesis to reconstitute normal tissue continuum at the pulp-dentin border [5].

An ideal scaffold for tissue regeneration would be made of biodegradable material with good mechanical strength. Each material offers a unique structure, chemistry, composition, and degradation profile [10]. The optimal degradation rate of substrate is a crucial factor for superior tissue regeneration. The materials used for hydrogel formation must degrade by time to release the contents and create space for new tissue formation. Ideally, the degradation rate should coincide with the rate of new tissue formation. Rapid degradation will cause scaffolds to lose their carrier function for cell growth, whereas a slow degradation rate can decrease the available space and impede new tissue formation [11].

Alginate forms a hydrogel by the interaction between G-blocks that associate to form tightly held junctions in the presence of divalent cations such as Ca^2+^, Sr^2+^ and Ba^2+^. The ionic crosslinked alginate hydrogel is usually weak and loses its mechanical integrity over time due to the reversible crosslinking and the outward flux of ions from the hydrogel [12]. Alternatively, the balance between resorption of dentin matrix and new dentin formation on matrix is critical for optimal dentin regeneration, and partially demineralized dentin matrix used in this study is thought to have optimal conditions [13].

In our previous publications [5, 14], we demonstrated that TDMH contributed to dentin regeneration and vital pulp conservation. However, to date, no study evaluated the physiological biodegradation of this novel injectable material. Hereby, we present complimentary data specifically focused on assessing homogeneity and mechanical properties of this novel scaffold and its biodegradability in vitro and in vivo when used in restoring dentin defect in DPC procedure.

## Methods

### Fabrication of human TDMH

TDMH was carried out as previously described by Holiel et al. [5]. The formed hydrogel was loaded into a single syringe to get rid of excess solution and get uniform injectable hydrogel mass [5, 14].

### In vitro* assessment of TDMH*

#### Homogeneity

To evaluate homogeneity of TDMH, three discs from the hydrogels were prepared using a cylinder with a height of 8 mm and a diameter of 10 mm then weighted adapted and used as follows: the dry weight to wet weight ratio of the hydrogel. The prepared hydrogel (n = 3) was cut horizontally to give four slices, which were numbered 1–4 (1 corresponding to the top slice and 4 to the bottom slice), each measuring 2 mm in diameter. These slices were weighed, dried to a constant weight, and then reweighed. The dry weight to wet weight ratio of the slices indicates the homogeneity of each gel. A homogeneous gel will have a consistent dry weight to wet weight ratio across its four constituent slices [15].

#### Mechanical properties

A total of three discs of dimensions 10 mm diameter × 8 mm height for TDMH were prepared to measure the compressive strength using a universal testing machine with a 30 mm diameter flat testing head. Before testing, hydrogels were allowed to equilibrate at room temperature for 72 h in buffer solution (pH = 7.4). Compression tests were carried out at a speed of 0.5 mm/min [16].

#### In vitro biodegradation

The degradation behavior of the prepared hydrogels was studied in phosphate buffered saline (PBS, Sigma-Aldrich, St. Louis, MO, USA), pH 7.4 under incubation for 2 months. Hydrogels discs of 10 mm diameter × 2 mm height were weighed by an electronic balance (RADWAG, AS 220.R2, Poland) before being incubated in 50 mL PBS. To maintain the PBS quality and avoid oversaturating the solution with degraded products, PBS was changed every 3 days [17]. On a weekly basis, the hydrogels were reweighed after removing excess surface solution by a filter paper (n = 3, analyzed in triplicate). This procedure was repeated until no further weight change was detected (equilibrium state). The percent weight change of hydrogels concerning time was determined by using the following equation: Q (%) = (W1 − W0)/Wo × 100.

Where Q is the percentage weight change, W0 is the initial weight of hydrogel before degradation and W1 is the weight of hydrogel after degradation [18, 19].

### In vivo* biodegradation of TDMH*

#### Study design

Twenty intact human premolars planned to be extracted for orthodontic reasons were selected in patients aged between 15 and 25 years. The minimal sample size was calculated based on a previous study aimed to evaluate the feasibility of PR-DDM as the scaffold for regenerating bone in critical-size iliac defects [20]. By adopting a power of 90% (β = 0.10) to detect a standardized effect size in percentage of new dentin formation (primary outcome) of 2.507, and level of significance 5% (α = 0.05) (Analysis of variance (ANOVA), F test was used, the minimum required sample size was found to be 4 teeth per group, increased to 5 to make up for histological processing errors. The total required sample size = number per timepoint × number of timepoints = 5 × 4 = 20. Sample size was calculated using G*Power (Version 3.1.9.2) [21]. Inclusion criteria were patients having four premolars with closed apices that required orthodontic extraction. Subjects were treated in accordance with the Helsinki declaration. Informed consents were obtained from subjects and/or parents (legal guardian) after experimental rationale, clinical procedures and possible risks were explained [5, 22]. All experimental procedures were reviewed and approved by the Institutional Ethical Committee.

#### Operative procedure

Occlusal cavities were prepared by using a sterile 245 bur (Komet, Hamburg, Germany) under complete rubber dam isolation. A standard pulpal exposure of 1–1.5 mm was made with round diamond burs, and hemostasis was obtained with cotton moistened with sterile saline. Exposed pulps were capped with TDMH, freshly mixed hydrogel was injected over the exposed pulp using a single syringe (5 mm chamber) leading to extrusion of a homogeneous composite to the site of defect. The residual material was carefully removed using a moistened cotton pellet. Then, the cavities in all groups were lined with a layer of RMGI cement (SDI, Bauswater victoria, Australia) and resin composite restoration (3M-ESPE, St. Paul, MN, USA) was then incrementally placed and light-cured. All restorative procedures were carried out by a single experienced operator in the department of conservative dentistry to standardize the procedure. Patients were asked about postoperative sensitivity or pain throughout the study period. Pulp sensitivity was assessed preoperative and before extraction using thermal testing (Endo Ice, Hygienic; Coltene/Whaledent AG, Switzerland) and electric pulp tester (Digitest II Pulp tester; Parkell Inc, Edgewood, NY, USA) [5]. The teeth were divided into 4 experimental groups (n = 5) according to extraction interval after 2-, 8-, 12- and 16-weeks using a computerized method (www.randomizer.org).

#### Histological and histomorphometrical evaluation

After extraction, the apical third of all teeth were sectioned to facilitate fixation in 10% formalin for 2 weeks, the specimens were then demineralized in a decalcifying solution containing 10% formic acid and were embedded in paraffin. 5 micron-thick serial sections in the linguobuccal plane of the paraffin-embedded teeth were stained with hematoxylin–eosin [22]. Coded samples were used throughout the study to avoid possible bias. By using light microscope (BX41; Olympus, Tokyo, Japan.) connected to a high-resolution video camera, samples were evaluated for histological and histomorphometrical evaluation.

The histomorphometric evaluations consisted of measuring the scaffold area and the newly formed dentin relative to the total measurement area. Each compartment was measured using Image J Software (Version 1.8.0) [23]. The average value was used as the mean percentage area of each group. A statistical analysis was conducted to compare the area of TDMH and newly formed dentin at 2, 8, 12 and 16 weeks after DPC procedure.

### Statistical analysis

Data were analyzed using IBM SPSS software package version 20.0. (Armonk, NY: IBM Corp). For continuous data, they were tested for normality by the Shapiro–Wilk test. Quantitative data were expressed as range, mean, standard deviation and confidence interval of mean. One way ANOVA test was used for comparing the four slices of TDMH and followed by Post Hoc test (Tukey) for pairwise comparison according to homogeneity. ANOVA with repeated measures was used to compare between more than two periods and followed by Post Hoc test (adjusted Bonferroni) for pairwise comparison according to in vitro biodegradation. While One way ANOVA test was used for comparing the different studied groups and followed by Post Hoc test (Bonferroni) for pairwise comparison according to in vivo biodegradation. Pearson coefficient was used to correlate between two normally distributed quantitative variables. Significance of the obtained results was judged at the 5% level.

## Results

### In vitro* assessment of TDMH*

#### Homogeneity

The homogeneity was determined by comparing the hydration degree of four slices of TDMH. Slices were weighted in the swollen state (Ws), dried and reweighted (Wd). Homogeneity was calculated as the average of the Wd/Ws ratios of the different discs of the hydrogel (Table 1). No statistical differences among hydrogel slices were detected with (*p* = 0.192)., indicating that the used method for preparing TDMH is effective in producing a homogenous hydrogel that makes the degradation behavior equal all over the hydrogel mass.

**Table 1:** Comparison between the different studied slices of TDMH according to homogeneity (%)

TDMH	Homogeneity (%)
	Mean ± SD
Slice 1	6.77 ± 0.25
Slice 2	6.17 ± 0.06
Slice 3	6.60 ± 0.17
Slice 4	6.07 ± 0.76
F	2.007
*p*	0.192

SD, Standard deviation, F, F for one way ANOVA test, *p*, *p* value for comparing between the studied slices

#### Mechanical properties

**Fig. 1:**
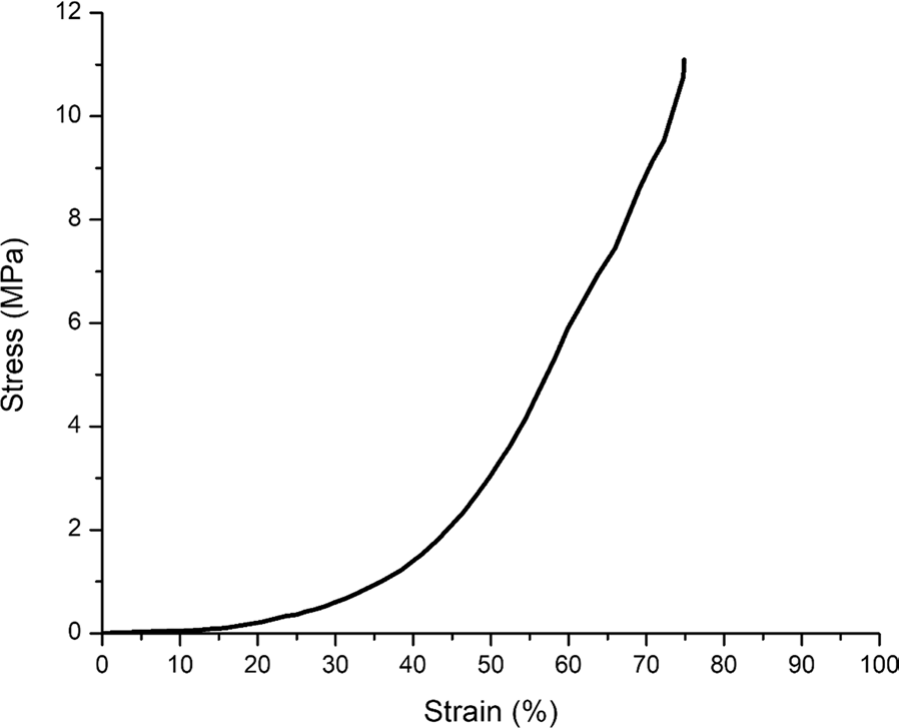
The stress–strain curve reflecting the deformation abilities/ stability of TDMH under stress

The compression test was used to characterize the compression moduli of TDMH. The result showed that the compression modulus of TDMH was (30.45 ± 1.11 kPa). The stress–strain curve reflected the deformation abilities/ stability of the hydrogels under stress. As shown in Fig. 1, the hydrogel exhibited significant stability and almost completely recovered after excessive compression.

#### In vitro biodegradation

**Fig. 2:**
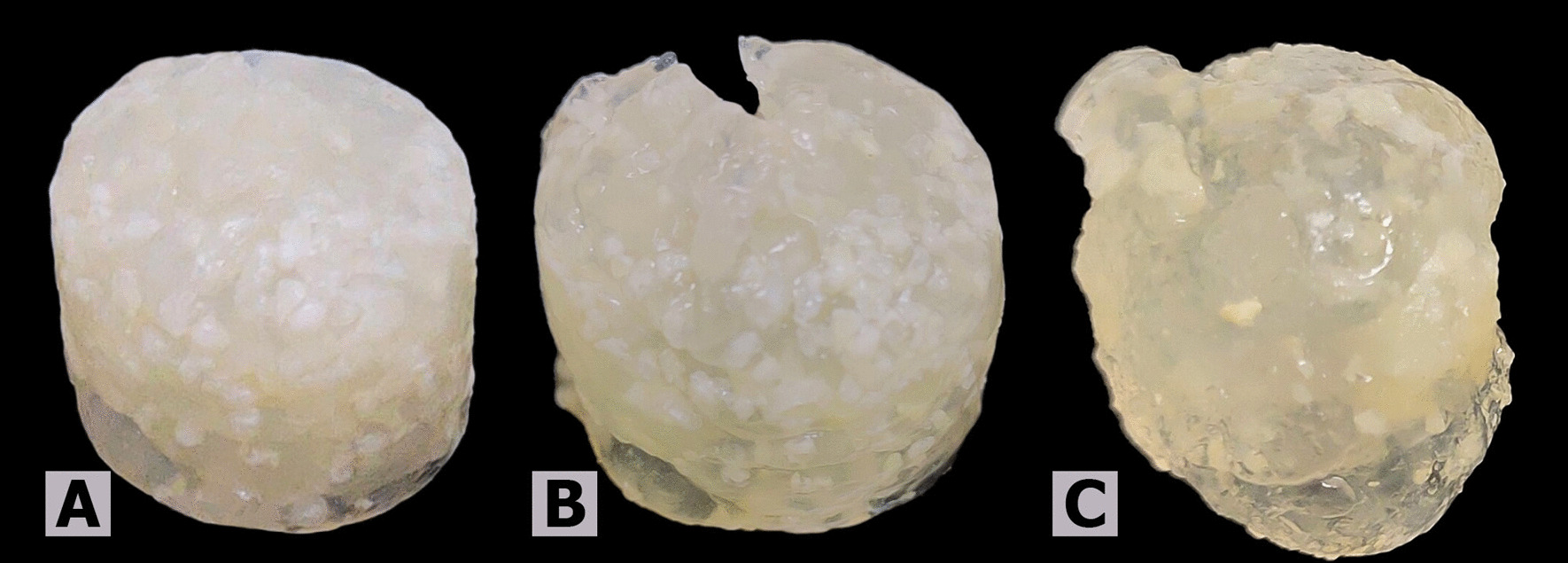
Degradation behavior of TDMH **A** at 1st week, **B** at 4th weeks, **C** at 7th week

**Fig. 3:**
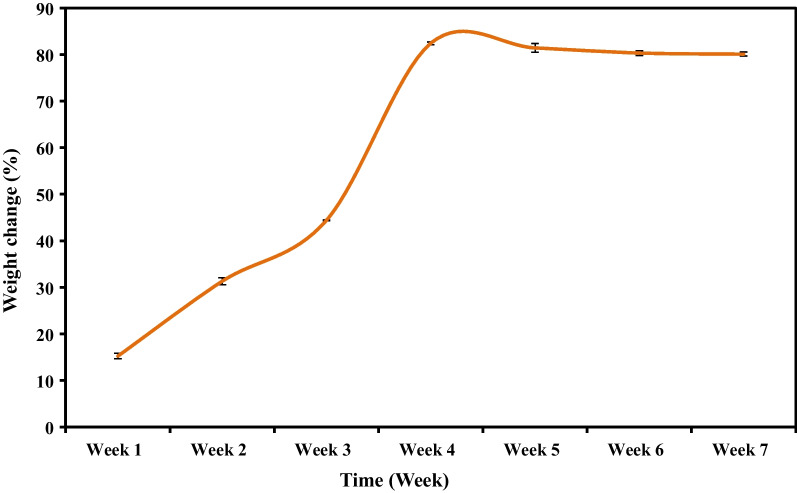
Percentage of weight change of TDMH

The in vitro degradation behavior of TDMH in PBS at pH 7.4 is presented in Fig. 2. TDMH retained their shape well up to 4 weeks, attained the equilibrium state at 5, 6, and 7 weeks, as they remained intact and maintained their bulk geometry with no further weight change (Fig. 3). Within 8 weeks, the hydrogel samples began losing structural fidelity, making it difficult to remove excess surface solution by a filter paper and palace them on the balance to be weighted (it was given a fault reading), and after that, they were completely degraded.

### In vivo* biodegradation of TDMH*

#### Clinical evaluation

Throughout the study, no symptoms were reported by the patients. All teeth were cold- and electrosensitive prior to extraction.

#### Histological and histomorphometrical evaluation

**Fig. 4:**
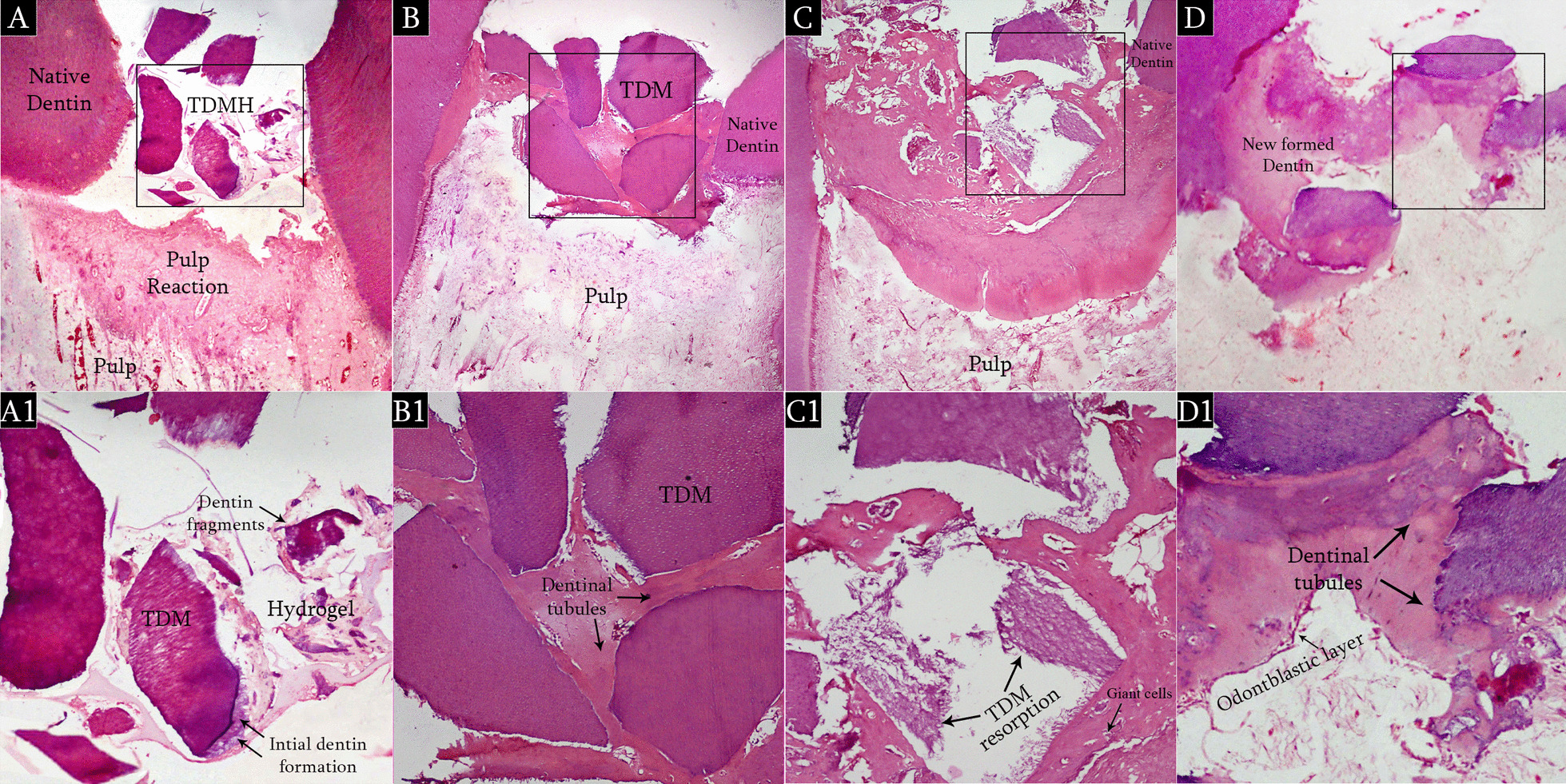
Light micrographs of human pulp capped with TDMH **A** After 2 weeks, scattered fragments of newly formed dentin seen along within the hydrogel, **B** After 8 weeks, hTDM completely incorporated in the newly formed dentin with numerous visible dentinal tubules. **C** After 12 weeks, hTDM resorbed during new dentin formation by giant cells with regular odontoblast layer. **D** After 16 weeks, thick continuous formed dentin completely spanning the distance between the two edges of the exposure site, with normal pulp morphology, × 100. (**A1**, **B1**, **C1**, **D1**) Higher magnification of boxed areas (**A**, **B**, **C**, **D**), × 200

Histological analysis after 2 weeks showed TDM scaffold with scattered fragments of newly formed dentin within the hydrogel (Fig. 4A, A1). After 8 weeks, TDM scaffold was completely incorporated in the newly formed dentin tissue with traversing dentinal tubules were evident in the formed dentin (Fig. 4B, B1). The cellular absorption of TDM was observed by giant cells at 12 weeks, which were attached with the scaffold. The absorption of TDM without the presence of giant cells was also seen in several areas. Interestingly, odontoblasts on the new dentin and giant cells on the absorbed scaffold appeared simultaneously (Fig. 4C, C1). While after 16 weeks, a thick continuous newly formed dentin completely spanning the distance between the two edges of the exposure site could be seen, with a significant reduction in TDMH area and a simultaneous significant increase in the new dentin area (Fig. 4D, D1).

**Fig. 5:**
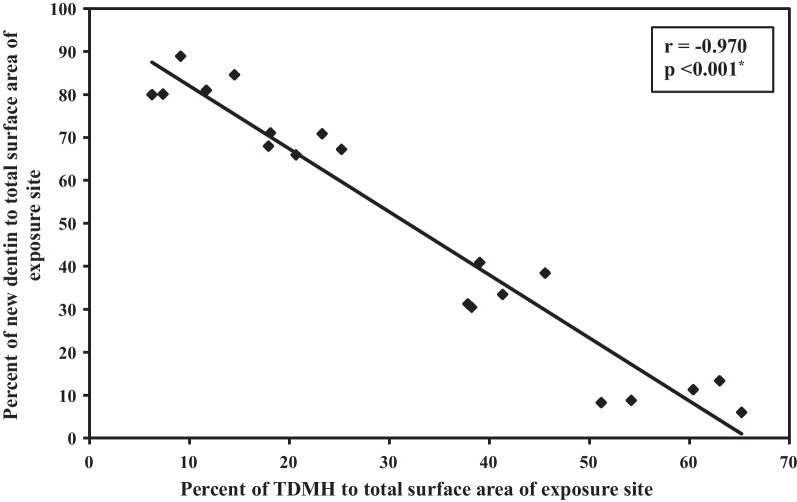
Correlation between percentage of TDMH and newly formed dentin area to total surface area of the exposure site in total sample

Histomorphometric measurements showed that the mean values of TDMH area fraction were on average 58.8% ± 5.9 and 9.8% ± 3.3 at 2 and 16 weeks, while the new dentin occupied 9.5% ± 2.8 at 2 weeks and 82.9% ± 3.8 at 16 weeks (Table 2). These results demonstrated that the ratio of TDMH decreased sequentially with the increase in new dentin area with a significant negative correlation between the new dentin area with TDM scaffold area as presented in Fig. 5. The overall findings of the histomorphometrical analysis suggested that the cellular phagocytosis and physiological absorption were responsible for the scaffold biodegradation and the new dentin was entirely induced and conducted by the 3D structure of TDM scaffold.

**Table 2:** Comparison between the different studied periods according to percentage of TDMH and newly formed dentin area to total surface area of the exposure site

	Extraction intervals				F	*p*
	2 Weeks (n = 5)	8 Weeks (n = 5)	12 Weeks (n = 5)	16 Weeks (n = 5)		
*Percent of TDMH to total surface area of exposure site*						
Mean ± SD	58.8^a^ ± 5.9	40.4^b^ ± 3.2	21.02^c^ ± 3.2	9.8^d^ ± 3.3	139.97*	< 0.001*
*Percent of new dentin to total surface area of exposure site*						
Mean ± SD	9.5^d^ ± 2.8	34.9^c^ ± 4.6	68.6^b^ ± 2.3	82.9^a^ ± 3.8	450.95*	< 0.001*

Means with common letters are not significant (i.e. Means with Different letters are significant)

*F*, F for One way ANOVA test, Pairwise comparison bet. each 2 groups was done using Post Hoc Test (Bonferroni). *p*, *p* value for comparing between the different extraction intervals

*Statistically significant at *p* ≤ 0.05

## Discussion

Regeneration of the dentin–pulp complex is of paramount importance to regain tooth vitality. The hybrid materials have undergone significant research to better mimic the physiological, biochemical, and physical cues of native tissues. At a minimum, the ideal biomaterial for tissue engineering needs to meet the following essential criteria: biodegradability, proper elastic modulus, and good biocompatibility [24, 25]. The present histological study was conducted under controlled conditions that used intact teeth in healthy patients to avoid the interference by confounding factors and by strict protocol for DPC procedure which included rubber dam isolation, disinfection of the operative field using 2% chlorhexidine-gluconate, and standardized pulp exposure size, as have been done in previous studies [5, 22].

The hydrogel scaffolds used for dentin–pulp complex regeneration should be degradable upon implantation to be replaced by newly formed tissues [26]. Ideally, the rate of scaffold degradation should be compatible with the rate of newly formed tissue. Too-rapid scaffold degradation can compromise its cell-supporting function, while a too slow degradation rate can hinder new tissue formation [11, 27]. Our previous study extensively focused on the dentinogenic properties of the novel injectable TDMH to restore normal tissues in dentin defect areas after DPC and revealed teeth treated with TDMH showed a positive trend to dentin regeneration [5]. However, the current study specifically investigated in vitro and in vivo degradation behavior of TDMH essential for new dentin regeneration and the various parameters involved in subsequent degradation of the hydrogels in the oral fluids, homogeneity, and the mechanical stability.

Sodium alginate (SA) is a common term used for a family of unbranched polymers composed of 1,4-linked β-d-mannuronic and α-l-guluronic acid residues in varying proportions, sequence, and molecular weight. Its gelation takes place when divalent cations (usually Ca^2+^), interact ionically with blocks of guluronic acid residues, resulting in formation of the three-dimensional network which is usually described by ‘egg-box’ model [28]. As the encapsulation method is mild, and done at room temperature in aqueous medium, several sensitive drugs, proteins, living cell, enzymes have been successfully released through SA hydrogels [29, 30]. Previous studies have confirmed TDM could release dentinogenesis related proteins that not only play vital role in the proliferation and differentiation of DPSCs into odontoblasts, but also form a network to form new dentin and regulate mineralization during dentin development and regeneration [31, 32]. As a result, TDMH based scaffold induced a natural biological regeneration to reconstitute normal tissue continuum at the pulp-dentin border.

Tissue engineering scaffolds require structural uniformity not just for uniform cell distribution but also for well-controlled material degradation. The diffusion of nutrients into all parts of the gel and the elimination of metabolic wastes are ensured by uniform pore size and distribution. If the structure is homogeneous, mechanical properties are more consistent across the hydrogel and between samples as concluded by Espona et al. [15]. The findings of the present study showed that TDMH was homogenous ensuring degradation behavior equal all over the hydrogel mass.

Moreover, adequate mechanical properties are essential for hydrogel scaffolds to provide support for the cells and withstand mechanical loading [33, 34]. The reduction in the mechanical strength and degree of crosslinking of the hydrogels may lead to a faster degradation rate after arriving at exposure site. So, they cannot reside for a sufficiently long time in the defect site. Matrix stiffness also affects the phenotype and differentiation pathway of mesenchymal stem cells (MSCs) [35]. Hydrogel mechanical properties are influenced by hydrogel composition, concentration, method of fabrication in addition to crosslinking density, porosity and hydrogel modification [36–38]. Decreased porosity and increased crosslinking density can be used to increase the material's mechanical properties, but these changes may compromise cellular response and degradability [33, 39]. Therefore, a balance should exist between hydrogel mechanical properties and degradability [40]. Alternatively, the current study revealed that TDMH ranged within the described native hard tissues elastic moduli values (25–40 kPa) [41, 42], validating this novel injectable scaffold as 3D matrix able to mimic the characteristics of native hard tissues that encouraged odontogenic/ osteogenic differentiation as increasing the calcium content and crosslinking density could allow tailoring the mechanical properties.

Regarding the in vitro degradation behavior of TDMH, after the hydrogels were immersed in PBS for 4 weeks, pores of the hydrogels swelled and became larger than those of the as-obtained hydrogels (week 0). After fourth week the hydrogels lost its porous structure and at the end of the degradation experiment (week 8), TDMH became not porous. While after 8 weeks, it was completely degraded. Shahriari et al. [43] studied the in vitro degradation of alginate scaffolds and concluded that the scaffolds generally maintained their channels and bulk geometry for at least 28 days. Moshaverinia et al. [18] investigated the degradation behavior of hydrogel based on oxidized sodium alginate with different degrees of oxidation in PBS at 37 °C showing more degradability of oxidized alginate hydrogels and after 4 weeks of storage in PBS, almost 50% of the initial weight of the alginate hydrogels has been lost. Other studies have performed cell attachment studies on alginate for up to 10 weeks and suggested techniques such as adding NaCl to preserve material integrity [44, 45]. Moreover, the hydrogel properties can be further regulated by multifunctional crosslinking molecules, which provide a wider range and tighter control over degradation rates, as demonstrated by Lee et al. [46]. In the current study, such degradation rate of TDMH may be attributed to the combined crosslinking of calcium ions to SA and the presence of TDM powder which acting as an inorganic crosslinker that could restrict the movability of the SA polymer chains and therefore slow down their degradation rates [47].

Furthermore, the histological findings in this study showed SA hydrogel was totally degraded after 8 weeks and replaced with newly formed dentin, leaving partial degradable TDM at the exposure site, after that it was significantly replaced by newly formed dentin. These findings correlate well with its in vitro degradation behavior for the first 8 weeks of degradation, in which SA hydrogel totally degrades after 8 weeks. This comes in accordance with Lee et al. [48] who concluded that ionically crosslinked alginate hydrogels disintegrate progressively in vivo due to the release of the divalent cations crosslinking the hydrogel into the surrounding media in exchange with the monovalent cations, such as sodium ions.

Higher dentin area induced by TDMH was observed at 16 weeks compared with 2 weeks after DPC. Moreover, histomorphometric measurements revealed a significant reduction in TDMH area fraction with a simultaneous increase in new dentin area with a significant negative correlation. Until now, the exact mechanisms of demineralized dentin in vivo degradation have not been fully clarified. Both enzymatic digestion and cellular phagocytosis are the dynamic processes involved in the organ absorption [49, 50]. Partially demineralized TDM used in this study is thought to have optimal conditions as biodegradable scaffold for dentin regeneration as reported by Koga et al. [13]. This could be explained by the partial demineralization of TDM leads to superficial decalcified dentin that expose the organic matrix with an inner core of mineralized dentin that could be enzymatically degraded. The exposed collagen matrices were found to be degraded by enzymes under different physiological and pathological conditions [50]. In vitro dentin bio-absorption by collagenase digestion was also successfully evaluated [10].

Additionally, mineralized tissues are known to be degraded by multinucleated giant cells via phagocytosis [51]. The cellular phagocytosis of TDM by giant cells was revealed in some histological sections in the present study. Thus, we hypothesize that TDMH in our study was degraded by cellular and enzymatic digestion altogether. This comes in accordance with those of Kabir et al. [10] who concluded that gradual absorption of the DDM by multinucleated giant cells and the presence of osteoblasts suggests active bone remodeling at the grafted site.

Nevertheless, the size and shape of dentin matrix particles are also important parameters that can be altered in order to tailor the degradation profile of the composite for a specific application. The current study showed that TDM with large dentin particles approximately 500 μm particles sized powder accommodating the defect size, successfully acted as a bio-absorbable scaffold. The histological findings clearly demonstrated significant bio-absorption of the TDMH at 16 weeks compared to 2 weeks. The loss of the structural integrity of the scaffold confirmed the physiological absorption. This comes in agreement with Koga et al. [13] and Togari et al. [52] who demonstrated that the larger the particle size of TDM, the more prominent the bone regeneration as smaller particles implanted in bone defects had more resorbability, and they may have been resorbed in vivo before the initiation of new bone formation. In contrast, Chen et al. [53] used hTDM as a paste for DPC with smaller particle size < 76 μm and concluded that TDM paste could achieve the dental pulp reparative procedure but with short follow up period and no emphasis on its degradability.

Newly formed dentin in the defect site was directly connected with TDM and native dentin. Altogether, the results proved that TDMH acted as a biodegradable scaffold, and absorption of TDM provided sufficient spaces for the newly generated dentin into defect areas after DPC procedure with degradation rate matched the rate of new dentin formation. This study highlights TDMH might contribute as a novel scaffold for dentin engineering. However, the use of different teeth for analysis (not a repeated measure), and the presence of a RMGIC liner that may influence cell fate and tissue formation could be addressed as limitations to the present study. Therefore, further research is recommended at earlier and later endpoints, and the applicability of the scaffold for large defects should be further studied. Cryopreservation of TDM scaffold is also recommended to overcome time-consuming preparation technique.

## Conclusions

TDMH was homogenous and exhibited significant stability and almost completely recovered after excessive compression. TDMH generally maintained their bulk geometry throughout 7 weeks. The in vivo response to TDMH was characterized by extensive degradation of the hydrogel and dentin matrix particles and abundant formation of new dentin. Higher dentin area induced by TDMH was observed at 16 weeks compared with 2 weeks after DPC. The degradation rate of TDMH matched the rate of new dentin formation.

## Data Availability

The datasets used and analysed during the current study are available from the corresponding author on reasonable request.
